# 

**Published:** 1986-02

**Authors:** 


**Editor**
Mr M. G. Wilson
*40 Coombe Lane, Bristol BS9 2BJ*
*Tel: 0272-682320*

**Assistant Editor**
Dr P. R. Goddard

**Secretary Editorial Committee**
Mr B. P. Jones,
*Medical Librarian, Bristol University*

**Correspondents**
Dr A. E. Adam
Dr J. D. Davies
Dr J. Jancar
Dr H. G. Mather
Professor G. M. Stirratt
Dr J. L. G. Thomson
Dr M. J. Whitfield
Professor G. K. Wilcock
Professor R. C. N. Williamson
Dr D. W. Wright

**BRISTOL MEDICO-CHIRURGICAL SOCIETY**

**President**
Dr J. Jancar

**Secretary**
Mr R. N. Baird,
*23 Old Sneed Park, Bristol BS9 1RG*

**Treasurer**
Dr N. C. Tricks,
*The Mill, Wrington, Bristol*

**Published by**
**Hermiston Publications,**
*BMA Scottish House,*
*7 Drumsheugh Gardens,*
*Edinburgh EH3 7QP*

**Typesetting by**
*Avon Communications Ltd., The Sion,*
*Crown Glass Place, Nailsea, Bristol BS19 2EP*

**Printed by**
*Scottish County Press, Sherwood Industrial Estate,*
*Bonnyrigg, Midlothian*

### BRISTOL MEDICINE

**Bristol Medicine acknowledges with gratitude the support and encouragement it receives from Bristol-Myers Pharmaceuticals**

Established 1883

### BRISTOL MEDICO-CHIRURGICAL JOURNAL

**FEBRUARY 1986**
**Volume 101 (i)**

Published bi-monthly and distributed free of charge to all doctors in the South West of England

**Contents**

**Editorial**

**Chronic Venous Ulcers of the leg**

S. J. Calder and D. J. Leaper

**Eruptive Vellus Hair Cysts**

C. E. H. Grattan and R. R. M. Harman

**Skins are simpler than you think**

J. L. Burton

**From our Correspondents**

**Bristol Medico-Chirurgical Society**

**From our Foreign Correspondent**

**Opening of the Monica Britton Exhibition Hall**

**Starting to Sail**

A. Roskovec

**Surgical Club of South West England**

**Bristol Medico-Historical Society?**

Proposal to form a new Society

**Letter to the Editor**

'What I know remains unknown,
Unless by me to others shown.'
*Lucilius (180–102 BC)*

### From Dr Ian McKee – Hermiston Publications

In Bristol and The West Country, in line with our policy of adapting to the local circumstances, we are trying out a new idea. You already have an extremely successful local journal, the Bristol Medico-Chirurgical Journal, but this was in danger of being caught in the familiar spiral of increased costs leading to increased subscriptions followed by a decrease in subscribers and a consequential further increase in subscriptions. Our plan is to link up with this journal, combining our production techniques with its successful editorial policy to produce a bimonthly free publication available to all medical practitioners in Bristol and the West Country. We mark the fact that this is a joint effort by incorporating the traditional title of the Journal and the title BRISTOL MEDICINE on the front page. It is particularly appropriate that Bristol–Myers Pharmaceuticals Ltd have generously agreed to sponsor the Journal and we look forward to a long and happy relationship.

It is our aim that all medical practitioners in the area, be they active or retired, should be given the opportunity to receive BRISTOL MEDICINE. In the next few months we will do our best to see that this is done as efficiently as possible but there will inevitably be a settling-in period. If you, or any of your colleagues are not receiving a personal copy of the publication please contact us and we will remedy the omission. As a general practitioner myself I am aware of the sense of professional isolation that can occur, both in rural areas and in the big cities. I share with Michael Wilson the hope that many new readers will contribute letters and articles in the future and help break down these barriers.

A few notes about the Society which founded this Journal may be of interest to those who see it for the first time. It was founded in 1874 with fifty members, at that time there were 140 medical men in Bristol which had a population of 196,000 and there were 448 hospital beds. Regular meetings have been held ever since without interruption by two World Wars. Initially, as now, papers were given, and there were discussions on chosen themes and case presentations. From its inception the Society was a 'club' at which doctors from Bristol and the surrounding area could meet together and it formed a bond between doctors in general practice, hospital doctors, doctors in the public service and others. This is still the most important function of the Society. In the early days it was perhaps the main source of postgraduate education, nowadays there are meetings, conferences, courses on all subjects aplenty and the Postgraduate Centres a constant source of further education. Accordingly meetings of the Society are designed less to educate than to interest, and the social aspect is enhanced by a buffet supper. Most of the meetings are held in the Postgraduate Centres, but there are visits with clinical meetings to hospitals or to local places of interest, as for example last year to the new Jenner Museum at Berkeley. The wider nature of the subjects of papers, this year for example Iris Murdoch is a speaker, encourages doctors to bring along their wives who may not be medically qualified. New members are usually recruited by existing members, but any doctor wishing to join who does not know who to ask should write to the Hon. Secretary, Mr Roger Baird who will be glad to find a proposer. The Society is particularly glad to welcome young members.

The undersigned would like to propose the formation of a new Society with an interest in the History of Medicine. They would like to see a small group all of whom would participate actively—each member being willing to give a paper from time to time. They suggest four meetings a year at which 3 to 4 papers might be given followed by a buffet supper. If there was a membership of 25 to 30 a member would be called upon to give a paper about every two years. The meetings could suitably be held at the Postgraduate Centres at Southmead or Frenchay Hospitals. Members need not necessarily be medically qualified—an interest in the History of Medicine would be the only qualification. Guests would be welcome and guest speakers could be invited from time to time. An annual visit to a place of historical interest could be arranged. Will anyone interested in joining such a society please write to the editor of this journal.

Michael Wilson, Joseph Sluglett

